# Orthodontic Perspectives in the Interdisciplinary Management of Pediatric Obstructive Sleep Apnea

**DOI:** 10.3390/children12081066

**Published:** 2025-08-14

**Authors:** Silvia Müller-Hagedorn, Véronique Abadie, Theodosia Bartzela

**Affiliations:** 1 Department of Orthodontics, Freiburg Interdisciplinary Center for Craniofacial Anomalies, Medical Center—University of Freiburg, Hugstetter Straße 55, D-79106 Freiburg, Germany; silvia.mueller-hagedorn@gmx.de; 2 Pediatric Maxillofacial and Plastic Surgery Unit, Necker University Hospital, APHP, 149, rue de Sèvres, F-75015 Paris, France; 3 General Pediatrics Unit, Referral Center for Rare Diseases “Syndrome de Pierre Robin et Troubles de Succion-Déglutition Congénitaux”, Necker University Hospital, APHP, Paris Cité University, 149, rue de Sèvres, F-75015 Paris, France; 4 Department of Orthodontics, University Hospital Carl Gustav Carus Dresden, Technische Universität Dresden, Fetscherstrasse 74, D-01307 Dresden, Germany

**Keywords:** obstructive sleep apnea, orthodontics, craniofacial growth, airway obstruction, functional orthodontic treatment, palatal expansion, mandibular advancement, orthodontic appliances

## Abstract

Pediatric obstructive sleep apnea (OSA) is a highly prevalent, multifactorial, and often underdiagnosed condition with significant consequences for cognitive and behavioral development. Early detection and timely multidisciplinary interventions are essential, particularly in children with craniofacial anomalies or syndromes associated with increased OSA risks, to prevent long-term complications. This narrative review explores the orthodontists’ role in the interdisciplinary management of pediatric OSA, focusing on early screening for craniofacial risk factors and implementing interceptive orthodontic interventions that support favorable airway development and growth modulation. Through early and frequent interaction with pediatric patients, orthodontists are well-positioned to identify clinical signs of airway-related abnormalities and craniofacial risk factors such as mandibular and maxillary retrognathism, maxillary constriction, and high-arched palatal vaults. Orthodontic interventions such as rapid maxillary expansion (RME), mandibular advancement, and myofunctional therapy may improve airway patency in selected cases. These approaches should be coordinated and integrated within the multidisciplinary team, including orthodontists, pediatricians, sleep specialists, ENT specialists, and speech-language pathologists. Furthermore, caregivers’ involvement and patients’ compliance are keys to success. Despite encouraging clinical observations, current evidence is limited by heterogeneity and a lack of long-term outcome data. Future research should prioritize well-designed prospective trials, explore the effectiveness of combined therapeutic strategies, and support the development of standard diagnostic protocols. Equally important is a stronger focus on early diagnosis and preventive measures to enhance patient outcomes and long-term treatment strategies. Integrating orthodontists into early OSA care is essential for optimizing outcomes and reducing long-term morbidity.

## 1. Introduction

Obstructive sleep apnea (OSA) is a sleep-related breathing disorder characterized by recurrent partial or complete upper airway obstruction despite ongoing respiratory efforts [1]. These episodes lead to disrupted breathing, impaired ventilation, and fragmented sleep patterns [1,2]. The OSA spectrum ranges from simple snoring to more severe manifestations, such as upper airway resistance syndrome and true obstructive sleep apnea syndrome [3].

The pathogenesis of OSA is multifactorial and not yet fully understood. Both anatomical and functional factors contribute to its development [3]. In children, upper airway obstruction is often associated with anatomical issues, primarily adenoid and tonsillar hypertrophy [4]. Additionally, skeletal constraints, such as mandibular retrognathia, a reduced bony nasopharynx, and a narrow maxilla, can further compromise the airway. The association between certain craniofacial anomalies and the severity of OSA underscores the pivotal role of anatomical factors in its pathogenesis [5].

Moreover, functional impairments, including deficits in neuromuscular control or underlying neurological conditions, may further compromise airway patency. These factors directly affect the tone and function of the pharyngeal dilator muscles, contributing to the complex pathophysiology of the disorder [6,7,8,9,10]. During wakefulness, individuals with OSA already rely on increased activity of the airway dilator muscles as a compensatory mechanism to maintain airway patency. However, with sleep onset, this augmented dilator muscle activity diminishes or is lost, leading to pharyngeal collapse. Therefore, the reduction in muscle tone during sleep onset is considered a crucial factor in the pathogenesis of OSA [11]. These anatomical and functional factors are modulated by additional variables, including body mass index (BMI), gender, medical conditions, and age [12].

OSA has wide-ranging implications for the pediatric population, significantly affecting cognitive and behavioral development, leading to long-term neurodevelopmental consequences. It notably impairs attention, memory, behavior, and academic performance. Its prevalence is particularly concerning among children with learning and behavioral disabilities, where it is estimated to be about six to nine times higher than in the general pediatric population. Due to its association with multiple health complications, OSA represents a serious public health concern [13].

Therefore, OSA in children should never be overlooked, and treatment should be initiated promptly after diagnosis. Comprehensive management requires close collaboration among pediatricians, otolaryngologists, sleep specialists, speech-language pathologists, and orthodontists to ensure coordinated, multidisciplinary care.

Orthodontists play a crucial role in the early identification and intervention of pediatric OSA. Through growth modification, orthodontists may optimize compromised airways and support long-term treatment outcomes. This collaboration is vital in ensuring effective and individualized care tailored to the patient’s needs [14].

While early orthodontic intervention is promising, the timing and extent of treatment remain the subject of debate. Some experts advocate for early interceptive management as early as 2–3 years of age, while others call for caution against overtreatment due to the absence of robust, long-term data.

Furthermore, sleep disorders in children, such as insomnia, sleep-related breathing disorders, central disorders of hypersomnolence, circadian rhythm sleep–wake disorders, parasomnias, and sleep-related movement disorders, are quite prevalent and often multifactorial. For example, OSA may coexist with insomnias or parasomnias. Therefore, a thorough preliminary assessment of sleep habits and associated disorders is essential to guide appropriate treatment and avoid under- or overtreatment. Hence, the need for routine sleep assessments underscores the importance of incorporating sleep screening during pediatric check-ups, especially in children who exhibit daytime symptoms [15,16].

There are several differences between pediatric and adult manifestations of OSA. Snoring is a common symptom in both children and adults. The peak prevalence of OSA occurs in children between 2 and 8 years of age due to adenotonsillar hypertrophy. In adults, obesity is the leading risk factor for OSA. While adult OSA is more prevalent in males, OSA affects both genders equally in children before puberty. In addition, OSA presents differently in children compared to adults, with distinct clinical features and consequences. While children exhibit behavioral issues such as hyperactivity disorders, emotional instability, aggressiveness, difficulty in concentration, poor academic performance, bedwetting, nocturnal sweating, and failure to thrive, symptoms such as daytime sleepiness and fatigue, morning headache, and memory impairment are more prevalent in adults.

The assessment of OSA in children is complicated by physiological and maturational changes in respiratory patterns that occur during childhood, which must be considered for an accurate diagnosis. Diagnosis is mainly clinical and can be undertaken with the help of sleep questionnaires (Pediatric Sleep Questionnaire). Nevertheless, overnight polysomnography is the gold standard for all ages to confirm and assess the severity of OSA, although it is more challenging to perform in children.

Furthermore, differences in polysomnographic scoring exist between children and adults. These differences stem from variations in sleep patterns and respiratory physiology, leading to the use of different scoring criteria. Severity classification of OSA depends on apnea-hypopnea index (AHI): In children, an AHI of 1–5 events per hour represents mild OSA, 5–10 events per hour represents moderate OSA, and more than 10 events per hour is classified as severe OSA. In contrast, in adults, an AHI of 5–15 represents mild, 15–30 moderate, and greater than 30 severe OSA.

Generally, children with OSA exhibit more significant oxygen desaturations than adults. Furthermore, children are more vulnerable to oxygen disruptions because of ongoing physical and neurological development. If oxygen levels drop significantly, even for a short time, this can have a more severe impact on their growth, cognitive function, and overall health than in adults. Delays in recognizing OSA in children are often related to the assumption that pediatric OSA mimics adult OSA [17,18,19,20,21,22].

In summary, pediatric and adult OSA differ significantly in their pathophysiology, clinical presentation, and diagnostic thresholds. While adult OSA is typically associated with obesity, extensive daytime sleepiness, and cardiovascular risk, pediatric OSA more often stems from adenotonsillar hypertrophy, craniofacial anomalies, or neuromuscular tone deficits and presents with behavior issues and growth disturbances. The AHI cutoffs are notably more stringent in children (AHI ≥ 1 for diagnosis), and the interpretation of sleep studies requires age-specific normative data. These distinctions necessitate the development of tailored diagnostic and therapeutic pathways.

Thus, this narrative review aims to provide a balanced perspective on the role of orthodontists in the interdisciplinary management of pediatric OSA, focusing on early diagnosis, craniofacial growth modification, and personalized treatment planning that aligns with current recommendations [23]. Moreover, it outlines current treatment strategies, addresses clinical challenges, and proposes future research directions. A further key objective is to promote effective communication and collaboration within the interdisciplinary team while identifying future research priorities to improve outcomes in this patient population.

## 2. Epidemiology and Risk Factors

It is estimated that 3–26% of children are habitual snorers [24,25,26], with 1.2–5.7% of the general pediatric population exhibiting OSA [27,28]. The peak incidence of OSA occurs between the ages of 2 and 8 years. In pre-pubertal children, OSA affects both genders equally [29]. However, after puberty, its prevalence increases in males [30]. Among adolescents seeking orthodontic treatment, up to 13.3% exhibit an increased risk of sleep-disordered breathing [31,32]. OSA is significantly more prevalent among specific pediatric populations with underlying risk profiles, like anatomical, neuromuscular, genetic, and environmental contributors.

OSA affects up to 36% of children with obesity [33], and the prevalence may exceed 60% in those who habitually snore [34]. Pediatric obesity is strongly associated with increased subcutaneous fat deposition in the neck surrounding the airway and fatty infiltration in the tongue. This reduces airway patency and contributes to OSA in children. Additionally, fat deposition around the thoracic, abdominal, and visceral regions decreases lung volume and oxygen reserve [35]. Notably, a 10% increase in body weight correlates with a 32% increase in the AHI [36].

Among children with Down syndrome [37], prevalence ranges from 30% to 63%, linked to midface hypoplasia, macroglossia, and generalized muscle hypotonia.

In craniofacial syndromes [38,39], such as craniofacial dysostosis (e.g., Treacher Collins syndrome, Goldenhar syndrome, craniofacial microsomia) [40] and syndromic craniosynostosis (e.g., Crouzon and Apert syndromes) [41,42], the prevalence of OSA ranges from 40 to 60% [43]. Similarly, children with Robin sequence [44] and cleft lip and palate [45] exhibit increased susceptibility. In all conditions associated with micrognathia, including secondary micrognathia related to temporomandibular joint ankylosis, trauma, or juvenile idiopathic arthritis, the prevalence of OSA increases with the severity of mandibular deformity [46,47,48]. Other syndromes associated with an elevated OSA risk include, among others, Prader–Willi syndrome [49], achondroplasia, and pycnodysostosis [50].

Neuromuscular conditions, including muscle tone disorders and dysfunction in central breathing control, further contribute to the development of OSA. Additionally, children with a history of prematurity or seizure disorders are more prone to OSA. Chronic upper airway inflammation, related to asthma, allergies, recurrent respiratory infections, or exposure to environmental factors (e.g., parental smoking, air pollution), also significantly increases the risk [51]. Among children with ADHD, the prevalence of OSA is elevated, with a risk increase of up to 62.5%. Sleep bruxism is also observed in up to 40% of these cases [52].

Ethnic variation in craniofacial morphology and airway anatomy influences the risk of OSA. For example, African populations with macroglossia and low tongue posture are at higher risk. Asian and Hispanic populations with maxillary or bimaxillary retrognathism demonstrate increased susceptibility [53,54]. Since craniofacial morphology and airway structure differ between ethnic groups, these factors must be considered when assessing OSA risk and planning treatment [55].

## 3. Craniofacial Growth and Functional Impact of Mouth Breathing

Craniofacial and dentofacial development is influenced by genetics [56] and environmental factors, including functional influences, such as breathing mode, tongue posture, and orofacial muscle tone [57,58]. Today, it is accepted that cartilage at the cranial base synchondroses is a major growth center and a critical determinant of craniofacial growth. According to the functional matrix theory (Moss et al.) [59], craniofacial and dentofacial growth occur in response to functional needs, such as mastication, swallowing, and breathing, and likely in response to the growth of the nasal cartilage [59]. Favorable dental arch growth depends on nasal breathing with a closed mouth posture and the tongue in contact with the palate, acting as a mold for development [60,61].

In addition, a short lingual frenulum results in restricted tongue mobility and an altered tongue position. Alterations of the lingual frenulum, such as a short frenulum, may contribute to orofacial dysfunction, mouth breathing, and underdevelopment of the maxillofacial skeleton due to impaired tongue function. Traditionally, the lingual frenulum was assessed by measuring free tongue length; however, contemporary assessments involve a functional classification of restricted tongue mobility, known as the Tongue Range of Motion Ratio (TRMR). The TRMR is defined as the ratio of mouth opening with the tongue tip placed at the maxillary incisive papilla to the maximum interincisal mouth opening, providing a functional measure of tongue mobility. Studies have demonstrated that restricted tongue mobility, as measured by the TRMR scale, was associated with increased odds of probable sleep bruxism. This suggests that tongue mobility, in association with frenulum length, rather than tongue size, is more relevant for the etiology of sleep bruxism [62,63].

Furthermore, mouth breathing bypasses the nose’s natural functions of humidifying, warming, and filtering the inspired air. As a result, airborne particles are more likely to enter the respiratory tract, increasing the risk of respiratory infections and contributing to oral dryness. Thus, mouth breathing, in turn, irritates the oral mucosa, leading to mucosal edema and further enlargement of the adenoids and tonsils. Additionally, the open-mouth posture causes a decrease in the contractile efficiency of the upper airway muscles, further contributing to airway collapse. Hence, mouth breathing initiates a vicious circle that promotes progressive narrowing of the upper airway. Other consequences include chronic hypoxemia and hypercapnia, which can lead to respiratory acidosis, increased water and energy loss, and a decrease in nocturnal growth hormone release. Considering orofacial functions, mouth breathing is very often associated with a visceral swallowing pattern, speech alterations, and reduced mastication efficiency [64,65,66,67].

**Figure 1:**
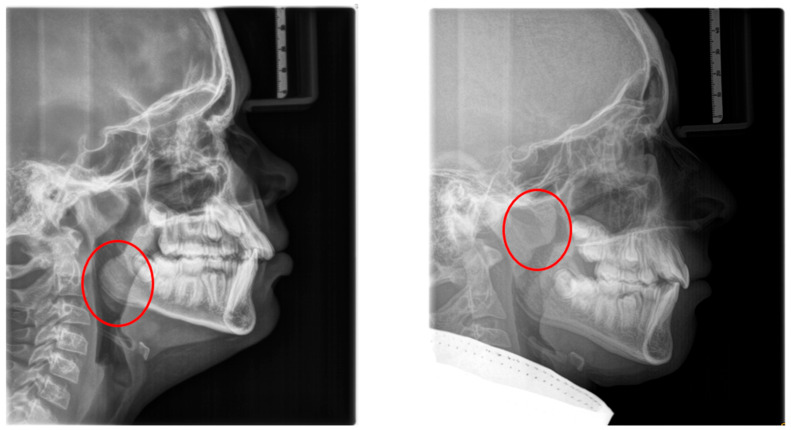
Radiographic lateral cephalograms: (**left**) red circle: enlarged tonsils; (**right**) red circle: enlarged adenoids.

Underlying these functional consequences, increased airway resistance, mainly caused by adenoidal hypertrophy, has been associated with craniofacial disharmony and malocclusion, as demonstrated by Linder-Aronson [57]. Mouth breathing has a multifactorial etiology, often stemming from anatomical obstructions such as adenotonsillar hypertrophy, midfacial hypoplasia, enlarged turbinates, or a deviated nasal septum. In some cases, it may also result from neuromuscular hypotonia or persist as a habitual pattern after treatment of anatomical obstructions (Figure 1).

**Figure 2:**
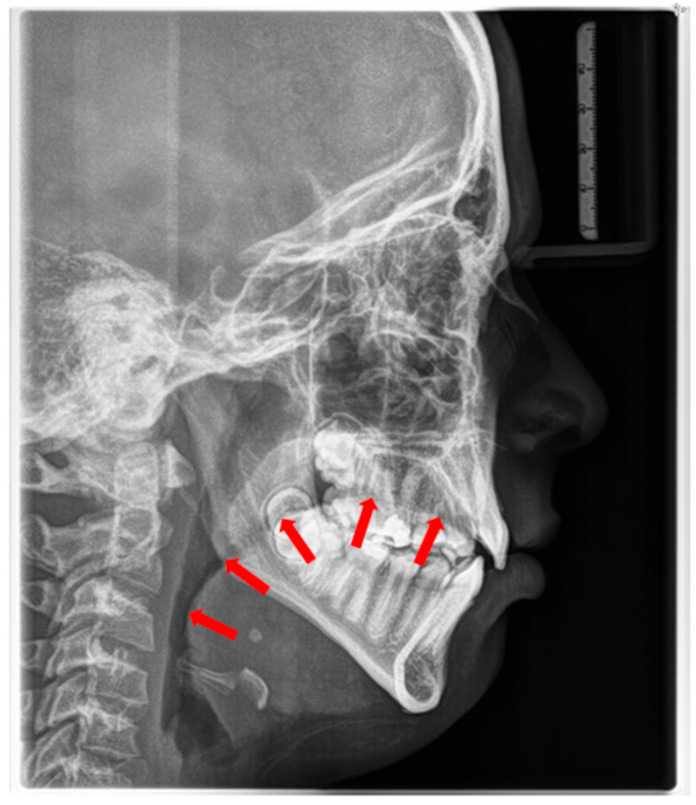
Radiographic cephalogram: Low tongue posture, the tongue protrudes into the oropharynx (red arrows indicate tongue shadow).

The soft tissue stretch theory, proposed by Solow and Kreiborg [68], postulates that mouth breathing alters head posture and muscle recruitment, thereby negatively affecting craniofacial growth. Mouth-breathing children often exhibit features collectively referred to as “adenoid facies” or long-face syndrome, along with reduced orofacial muscle tone [57,61,69,70,71]. A low tongue position is most often a result of mouth breathing (Figure 2).

This results in insufficient internal tongue pressure on the palate and a predominant cheek pressure, leading to a reduction in transverse growth of the maxilla and the development of a high-arched palate and lateral crossbites [72].

Importantly, some craniofacial adaptations related to mouth breathing may be partially reversible in young children [73]. These findings have been demonstrated in animal studies using Rhesus monkeys with experimentally induced nasal obstruction [74,75,76].

## 4. Craniofacial Anatomy in OSA Patients

Craniofacial anatomy is considered an underlying risk factor contributing to the development of OSA. Cephalometric abnormalities in patients with OSA were initially described by Riley et al. [77] and Guilleminault et al. [78]. Notably, craniofacial morphology observed in adults with OSA often resembles that of children with OSA or mouth breathers [79]. However, altered craniofacial anatomy might represents a physiologic compensation to the underlying clinical condition rather than a primary causative factor [80].

Cephalometric evaluation of patients with OSA has revealed several skeletal (a) and soft tissue (b) morphological alterations compared to normative values [81,82] (Table 1).

**Figure 3:**
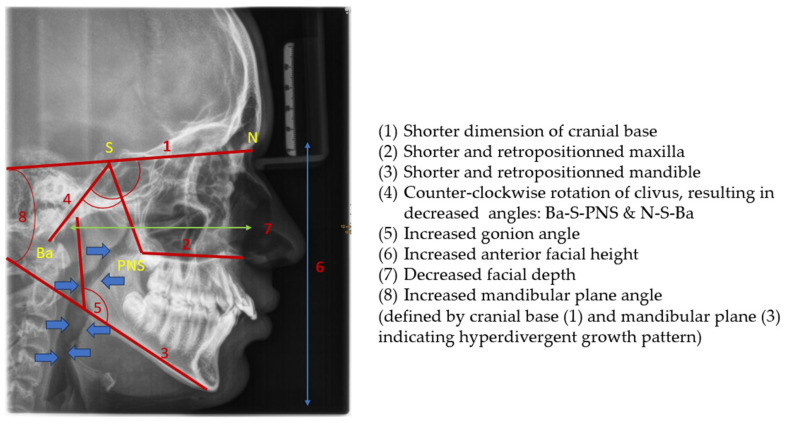
Radiographic lateral cephalogram: Typical craniofacial morphology in OSA patients. Blue arrows indicate the posterior airway space (PAS).

The consequences of these anatomical alterations result in a reduced size of the bony nasopharynx and a hyperdivergent facial pattern, characterized by increased anterior facial height. A bimaxillary retrognathic pattern, more pronounced in the mandible than the maxilla, and a decreased facial depth further contribute to a narrowed posterior airway space [83]. Together, these structural changes significantly reduce upper airway volume, thereby predisposing affected individuals to OSA (Figure 3).

## 5. Clinical Characteristics in Children with OSA

### 5.1. Extraoral Findings

**Figure 4:**
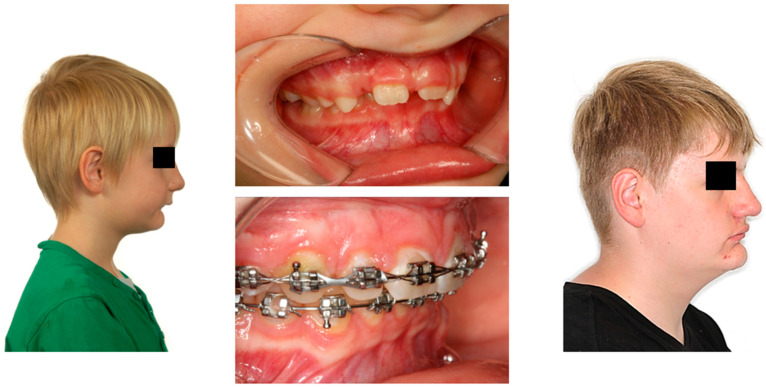
(**Left**): Extraoral photograph of a patient with OSA before treatment, with a convex profile and a retrognathic mandible; (**middle**): intraoral photographs of the same patient before and during treatment; (**right**): extraoral photograph at the end of treatment showing the mandibular advancement (Photographs shown with parental and patient’s consent).

These patients often exhibit “adenoid facies” characteristics and present with dark circles around the eyes, flattened cheekbones, dry lips, an open-mouth posture, a lowered mandibular posture, a low tongue position, labial incompetence, underdeveloped nasal bones, pronounced nasolabial furrows, which collectively complete the typical appearance [57,61,69,70,71]. They often present a convex profile due to a retrognathic mandible (Figure 4) and an increased mandibular angle. The lower facial third is frequently longer than the average (long-face, dolichofacial morphology) [69] (Table 2). Additionally, they exhibit an altered head position resulting from hyperextension of the cervical spine and an overall reduction in orofacial muscle tonicity.

### 5.2. Intraoral Findings

Malocclusion is highly prevalent in children with OSA. They often present posterior crossbites in addition to lateral functional shifts due to a narrow maxilla [84,85,86]. Regarding the palatal vault, it is higher and narrower than in non-affected children [87]. This is related to the altered equilibrium between the tongue and cheeks [72]. In addition, an anterior open bite and sometimes a deep bite, an increased overjet due to a retrognathic mandible, protruded upper incisors, and crowding in the maxilla and the mandible are constant findings [69,88,89].

**Figure 5:**
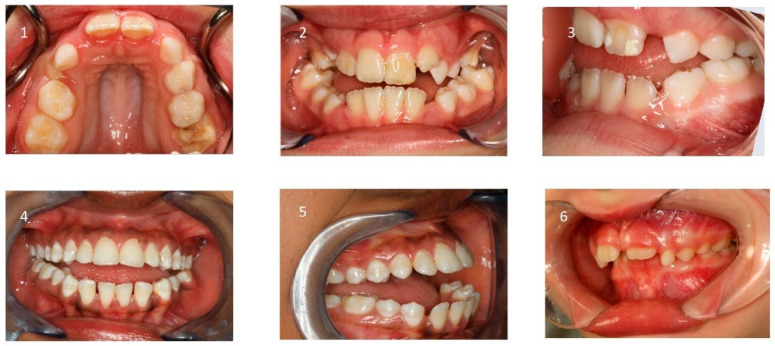
Intraoral findings: (1) narrow maxilla; (2) bilateral posterior crossbites, (3) low tongue position, (4) circular open bite; (5) protruded incisors; (6) deep bite and increased overjet.

Furthermore, ankyloglossia and reduced tongue mobility may be present [90,91,92,93]. (Figure 5 and Table 2).

### 5.3. The Typical “At-Risk Patient”

The typical “at-risk patient” exhibits [94] intraoral and extraoral features that should alert health professionals to initiate appropriate diagnostic evaluation (Table 3).

## 6. The Role of Orthodontics in OSA Diagnosis

### 6.1. Role of the Orthodontist in the Multiprofessional Team

All healthcare professionals involved in pediatric care, including pediatricians, ENT specialists, child psychiatrists, psychologists, speech-language pathologists, orthodontists, and sleep specialists, are called upon to screen patients for OSA. The pediatrician plays a central and guiding role in this setting. In cases of positive screening for OSA, children are referred to their pediatrician for further evaluation. If it is deemed necessary, the children are subsequently referred to a sleep specialist. Once OSA is diagnosed, treatment decisions are typically made by the pediatrician, in collaboration with the sleep specialist, prioritizing the most effective, evidence-based interventions. First-line treatment represents adenotonsillectomy in many cases.

If mouth breathing persists postoperatively, referral to a speech-language pathologist is indicated. In children with craniofacial anomalies, referral to an orthodontist is warranted. The orthodontist, in close collaboration with the speech-language pathologist, addresses the craniofacial disharmony (e.g., maxillary constriction, retrognathic mandible) and underlying myofunctional disorders. During orthodontic treatment, regular screening for OSA is essential, particularly in cases involving mandibular retrognathia.

Upon completion of treatment or in the event of clinical deterioration, patients are referred back to their pediatrician, who will assess the need for further referral to a sleep specialist. Close communication and ongoing information exchange among team members are critical to the success of care. The management of pediatric OSA is a collaborative, interdisciplinary effort that ensures comprehensive treatment, minimizes risks, and optimizes outcomes.

The orthodontist plays a critical role within the multidisciplinary team (e.g., ENT, pediatrics, sleep medicine) by assessing craniofacial structures that may contribute to OSA.

In fulfilling this role, the orthodontist’s contributions include the following:

(1) Collaborative assessment: The orthodontist works with other healthcare providers to assess craniofacial structures that may contribute to pediatric OSA.
(2) Diagnostic referral: Upon detecting signs of sleep-disordered breathing, the orthodontist refers the patient to a physician for a definitive diagnosis.
(3) Airway-focused treatment: The orthodontist may initiate treatment to address skeletal discrepancies and myofunctional disorders that contribute to airway narrowing.
(4) Caregiver education: Orthodontists inform caregivers about the potential risks of untreated OSA and the role of orthodontic therapy in improving airway function.
(5) Ongoing monitoring: The orthodontist continues to monitor patients’ craniofacial development and collaborates with the multidisciplinary team to ensure comprehensive care.

### 6.2. Screening for OSA

When evaluating children for potential sleep-related breathing disorders, several behavioral and clinical indicators should be considered. Key factors include previous diagnosis of OSA, habitual snoring, nasal obstruction, mouth breathing, and witnessed pauses in breathing during sleep. Additional signs such as sleep bruxism [95], abnormal sleep positions (e.g., hyperextension of the neck), and difficulties waking up in the morning, may also be present. Anthropometric data, such as height, weight, and body mass index (BMI), can offer further insights, as can neurodevelopmental indicators like developmental delays, poor academic performance, attention difficulties, and hyperactivity disorders. Behavioral concerns, including aggressive behavior, inappropriate bedwetting, may also signal underlying sleep disturbances. A thorough review of current medications and general sleep-related complaints-such as morning headaches, daytime sleepiness, or falling asleep quickly-should also be part of the assessment. For screening purposes, the Pediatric Sleep Questionnaire (PSG) is a valuable tool with high diagnostic value. It comprises 22 items addressing three major symptom domains: snoring, excessive daytime sleepiness, and inattentive or hyperactive behavior [96,97].

A well-designed screening should include a comprehensive orthodontic examination. In addition to the above-mentioned extra- and intraoral features, oral functions such as breathing and swallowing patterns, tongue size, function, rest position, speech, temporomandibular joint disorders, and the size of the tonsils should be evaluated. Compared to a control group, mouth-breathing children with OSA exhibited differences in oral microbiota, higher acidity, and poorer dental status [98].

A comprehensive “Pediatric Obstructive Sleep Apnea Diagnostic Examination Form” was recently developed by a German working group. Drawing upon both clinical experience and the current literature, the form integrates craniofacial and functional assessment items relevant to pediatric OSA [99]. This form comprises two pages and provides a structured, comprehensive evaluation of craniofacial and functional characteristics associated with the condition. It is self-explanatory and suitable for use by all health professionals involved in the diagnosis and treatment planning of pediatric OSA.

### 6.3. Aims of Orthodontic Treatment

Orthodontic treatment plans for patients with OSA should follow established principles used for treating dental and skeletal deformities, just as for those not affected by OSA. Treatment planning must consider both anatomic and functional risk factors for OSA that compromise airway volume. The treatment goal is to ensure a sufficiently large intraoral space and maintain proper tongue posture against the hard palate. Orthodontic devices should be used only in patients with specific structural issues, such as a narrow maxilla, and combined with myofunctional therapy, if necessary.

Orthodontic treatment can mitigate or even treat OSA by correcting the underlying skeletal disharmony and enlarging the upper airway. Treatment should align with growth phases and guide development towards a favorable skeletal and functional pattern. Thus, orthodontic treatment may be curative or at least reduce the symptoms of OSA and may even prevent the onset of OSA in later life.

Once growth is complete, underlying skeletal disharmony can only be corrected through orthognathic surgery and surgically assisted rapid maxillary expansion [100].

## 7. Imaging and Radiologic Assessment

**Figure 6:**
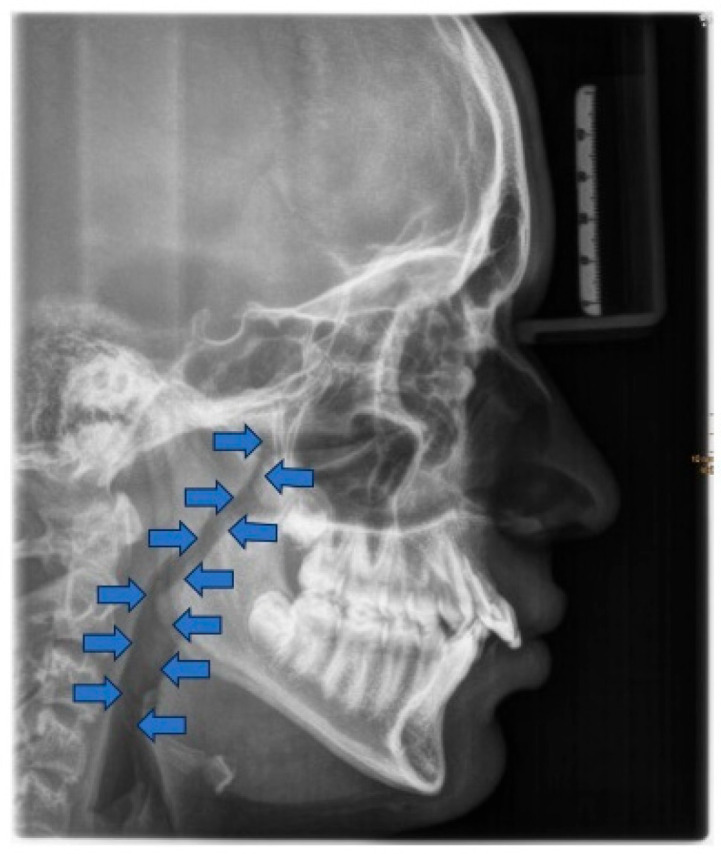
Radiographic cephalogram: Blue arrows indicate the posterior airway space.

Lateral cephalometry allows for systematic assessment of craniofacial structures, including both hard and soft tissues. Furthermore, it enables the evaluation of the sagittal dimension of the posterior airway space (PAS) and can therefore serve as a screening tool [101,102]. This diagnostic tool is reproducible, affordable, easily accessible in an orthodontic office, involves minimal radiation exposure, and is non-invasive [2] (Figure 6). The cephalometric radiographs are taken in an upright and natural head position, where the eyes focus ahead with a horizontal visual axis parallel to the floor (Frankfort horizontal plane). The occlusion should be the habitual bite (not forced into maximum intercuspation) and the lips in gentle contact (not forcefully closed).

Nevertheless, it is essential to note that the soft tissues in the upper airway behave differently when a person is asleep, in a supine position, compared to an upright position [103,104,105,106]. Several studies attempted to establish a relation between airway dimensions and craniofacial structures in subjects with OSA through cephalometric assessment [107,108,109].

However, a comprehensive assessment of the airway is better achieved with cone-beam computed tomography (CBCT). This three-dimensional evaluation offers a detailed visualization of the airway and the surrounding structures [110]. Though no universally accepted airway volume threshold exists to predict OSA risk, patients with OSA generally have smaller airway volumes compared to their unaffected counterparts [111]. No radiographic methods have been reported to have high specificity and sensitivity, serving as actual risk-assessing tools for OSA [100]. Moreover, the challenges of studying a functional airway using static images are inherently limited [112].

In contrast, drug-induced sleep endoscopy (DISE) is a technique used to evaluate the airway under sleep-like sedation. It is considered the most reliable method for assessing functional airway obstruction and is typically reserved for specific diagnostic questions to guide individualized treatment planning [113].

## 8. Craniofacial and Orthodontic Treatment Strategies for Pediatric OSA

Given the growing recognition of craniofacial contributions to OSA, orthodontic treatment has become an increasingly relevant component of multidisciplinary management. This section presents orthodontic treatment options for pediatric OSA across the full developmental spectrum from birth to adolescence. Pediatric OSA affects a significant proportion of children, particularly those with craniofacial anomalies or neuromuscular conditions. When identified early, many craniofacial and functional discrepancies can be addressed conservatively through growth-guided functional orthodontic interventions. In contrast, once growth is complete, only surgical options, such as orthognathic surgery and surgically assisted maxillary expansion, are effective in correcting the underlying skeletal deformity of OSA patients in a curative way.

This section provides an overview of the various functional orthodontic treatment modalities that can be employed to manage pediatric OSA during growth, supporting airway development, and reducing the severity or progression of pediatric OSA.

### 8.1. Prevention

Abnormal oral habits play a significant role in the development of malocclusions in children. Habits such as thumb and finger sucking, prolonged pacifier use, mouth breathing, a persistent infantile swallowing pattern, and low tongue posture exert abnormal forces on developing dentition and craniofacial structures. Management of these habits involves behavioral modification, child education, and parental counseling [114].

While sucking habits are considered acceptable during infancy, their persistence beyond 2–4 years of age, particularly into the mixed dentition stage, raises concern due to their potential impact on craniofacial and dental structures. Insofar as sucking habits, mouth breathing, malocclusions, low tongue position, and further oral dysfunctions are interdependent factors [94] and may interfere with craniofacial and dental development, they should be addressed early. If left untreated, these factors may also contribute to the development of OSA.

From a preventive perspective, promoting nasal breathing and eliminating these habits at an early stage are essential. Myofunctional therapy should be initiated to restore proper orofacial function, including optimal tongue posture and normal breathing and swallowing patterns. Early interception of oral habits, combined with restoration of nasal breathing and targeted myofunctional therapy, constitutes a proactive strategy for preventing craniofacial growth disturbances and potentially reducing the risk of developing OSA.

### 8.2. Neonatal Intervention: Robin Sequence

Robin sequence is a malformative triad characterized by mandibular micro- and retrognathia, glossoptosis, and upper airway obstruction. A U-shaped cleft palate may be present, although it is not a consistent finding [115]. A birth prevalence of 12.4 per 100,000 live births has been reported, classifying Robin sequence as a rare disease [116]. Robin sequence may occur as an isolated entity, as part of a syndrome, or in association with other malformations. Several early treatment options exist to address upper airway obstruction in affected neonates. These range from less invasive measures, such as prone positioning, nasopharyngeal airway placement, non-invasive CPAP ventilation, and the use of a Tübingen palatal plate (TPP) [117,118], to more invasive surgical treatments, including mandibular distraction osteogenesis [119,120] or tracheostomy. The TPP has demonstrated its effectiveness in neonates with isolated and syndromic Robin sequence [117,118].

**Figure 7:**
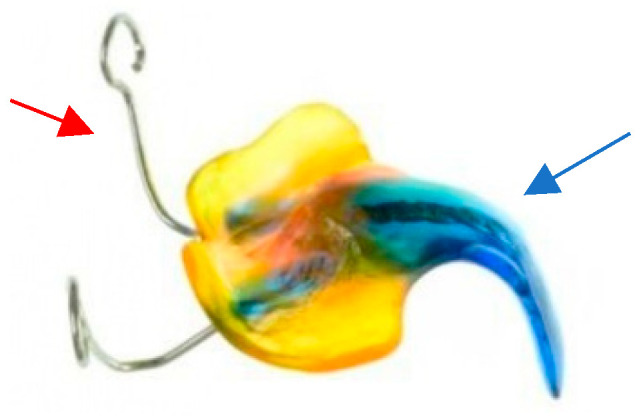
Tübingen palatal plate for neonates with Robin sequence. The main body of the appliance is constructed from colored acrylic. The spur is consistently made in a dark color to facilitate identification during endoscopy. A safety wire is integrated into the spur (blue arrow) to safeguard the device against mechanical failure. Two extension wires (red arrow) are used for the stabilization of the appliance on the patient’s face.

TPP consists of a palatal part and an attached spur (Figure 7). The spur repositions the tongue anteriorly and horizontally, thereby enlarging the pharyngeal airway (immediate effect). Functioning as a functional orthodontic appliance, it also promotes condylar growth, leading to catch-up growth of the micrognathic mandible over time (long-term effect) [121]. TPP treatment is accompanied by myofunctional therapy and feeding training.

### 8.3. Myofunctional Therapy

Myofunctional therapy aims to reduce the frequency and severity of pediatric OSA and snoring [122,123] by improving labial seal, lip tone, and nasal breathing while promoting favorable tongue positioning within the oral cavity. By re-establishing tongue-to-palate contact, a stable posterior oral seal [124] is created, preventing posterior displacement of the tongue during sleep. Additionally, respiratory muscle therapy enhances coordination between the upper and lower airway muscles, thereby improving airway patency. Patients are instructed to perform daily orofacial exercises to strengthen the tongue and orofacial muscles and promote correct tongue posture (active myofunctional treatment). A recent systematic review and meta-analysis (SR-MA) supports the role of oropharyngeal muscle therapy as adjunct management of pediatric OSA, showing improvement in key respiratory parameters, such as the AHI and apnea index (AI), patient-reported outcomes (Epworth Sleepiness Scale, Pittsburgh Sleep Quality Index), and snoring frequency [125]. Although these exercises are well-tolerated and straightforward, patient cooperation and adherence are crucial for any potential benefits associated with this type of treatment [126]. Several studies have also proposed passive myofunctional treatment using intraoral appliances as an alternative or adjunct to active exercises [127,128].

### 8.4. Interceptive Functional Appliances and Screening Devices

#### 8.4.1. Interceptive Functional Appliances

**Figure 8:**
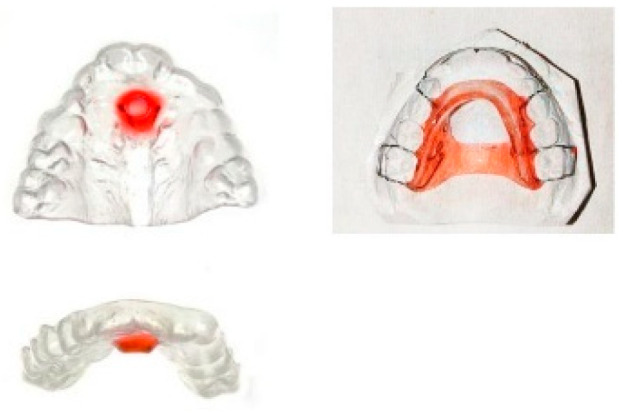
Custom-made myofunctional appliances. The red component of the appliances on the left side serves as a stimulation element for the tongue.

Interceptive functional appliances aim to promote proper tongue posture by stimulating the tongue to rest directly behind the upper incisors, thereby improving habitual tongue posture (Figure 8).

In a study, an oral device incorporating a tongue stimulation element demonstrated improvements after 12 months of treatment, including enhanced nasal breathing during sleep, mandibular linear growth, improved airway morphology, and increased patient-reported quality of life [129].

Recently, new prefabricated myofunctional devices have been introduced to support oral habit correction and functional training. The “Froggy Mouth^®^” device has demonstrated efficacy in correcting atypical swallowing patterns and eliminating dysfunctional oral habits [130].

Another prefabricated myofunctional appliance, the “Myobrace^®^” device, is designed for cessation of habits, occlusal guidance, and orofacial muscle training. This one appears particularly beneficial in managing Class II Division 1 malocclusions [131].

#### 8.4.2. Screening Devices

The vestibular plate is a functional appliance designed to eliminate abnormal perioral forces and support the undisturbed development of the orofacial system, particularly during the deciduous and early mixed dentition stages. It primarily influences the function of lip, cheek, and tongue muscles to counteract the deformative effects of soft tissue dysfunctions, such as tongue thrust, low tongue posture, and habitual mouth breathing. The appliance may help eliminate sucking habits.

In deciduous dentition, the vestibular plate helps correct acquired malocclusions resulting from abnormal habits and mouth breathing, such as an anterior open bite associated with persistent finger sucking and retained infantile swallowing patterns. In mixed dentition stage, these appliances may be used as adjuncts before comprehensive orthodontic treatment to reduce abnormal perioral muscular interferences.

This approach is most effective when implemented as part of an early interceptive treatment. Additionally, myofunctional exercises are often helpful during screening therapy.

**Figure 9:**
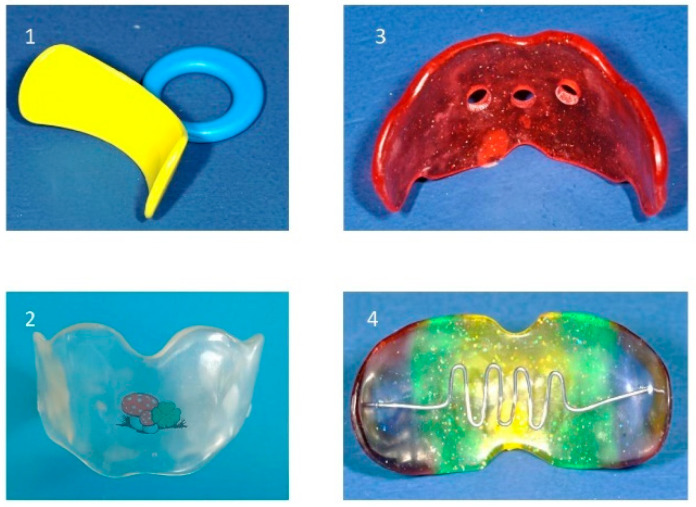
Various types of vestibular screens: (**1**) prefabricated screen, (**2**) custom-made screen, (**3**) screen with breathing holes, (**4**) screen with tongue crib.

The vestibular plate may also promote anterior mandibular positioning. In cases of tongue dysfunction, additional elements, such as a tongue crib or shield, can be integrated to enhance treatment. Breathing holes can be progressively reduced to facilitate the transition from habitual mouth breathing to nasal breathing. This is particularly important, as some children continue to mouth breathe even after adenotonsillectomy, which may increase the risk of adenoidal regrowth. Thus, the vestibular screens are generally custom-fabricated but also available in prefabricated designs (Figure 9).

### 8.5. Maxillary Expansion

Transverse maxillary deficiency requires skeletal maxillary expansion, which is achieved by stimulating growth through separation at the mid-palatal suture. As individuals age, the mid-palatal maxillary suture becomes increasingly interdigitated, increasing its resistance to expansion.

In adolescence, expansion may involve fracturing of the bony interdigitations, which can reduce both the extent and stability of expansion. Therefore, maxillary expansion is ideally performed at an early age when skeletal responsiveness is optimal [132].

The maxillary expansion protocol is primarily determined by the patient’s age and the stage of maturation of the mid-palatal suture. In preschool children up to approximately 8 years of age, the mid-palatal suture remains highly malleable. These patients can benefit from removable expanders or slow fixed maxillary expanders that apply light and gradual forces to achieve skeletal expansion.

For patients aged 15 years or younger with a maturing mid-palatal suture, conventional fixed rapid maxillary expansion (RME) appliances are an effective treatment option. These devices facilitate predictable skeletal expansion before complete suture fusion occurs.

In older adolescents and young adults with advanced suture maturation, mini-implant/mini-screw-assisted palatal expansion (MARPE) is the preferred treatment option. This technique anchors the expander to the palate using mini-implants, allowing manual activation to split the mid-palatal suture. MARPE minimizes side effects, such as buccal bone loss around anchor teeth and alveolar bone tipping, while primarily promoting skeletal expansion.

In skeletally mature adults with a fully fused mid-palatal suture, only surgically assisted palatal expansion (SARPE), distraction osteogenesis maxillary expansion (DOME), or other osteotomy-based techniques are effective. These procedures involve surgically weakening or segmenting the maxillary bone to enable orthopedic expansion [133].

#### 8.5.1. Removable Expanders

**Figure 10:**
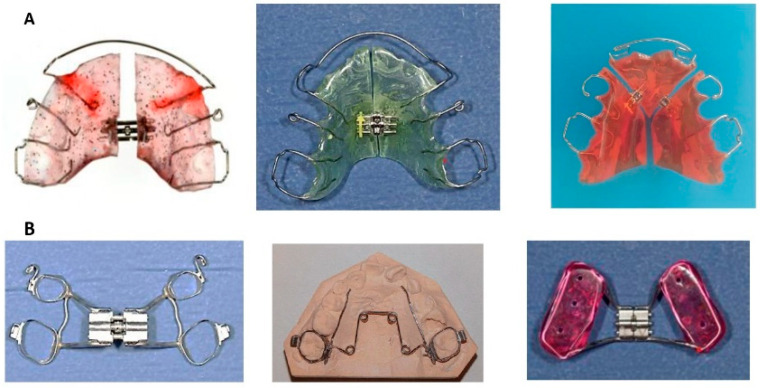
(**A**): Removable maxillary expanders, (**B**): Fixed maxillary expanders: Left: Hyrax-Expander, middle: Quad-Helix, right: Bonded expander.

These appliances are designed for slow maxillary expansion. They are mainly indicated in cases of unilateral, bilateral, or localized dental arch expansion. In young children, expansion remains predominantly skeletal, whereas in late adolescence the effects are mainly dental. Palatal expansion protocols necessitate meticulous control to prevent dislodging of the appliance. The activation of the screw should not exceed one turn every 5 to 7 days. In the case of a high palate, up to 1 turn (90°) every 5 days is possible (Figure 10). Patient compliance is crucial, as the appliance requires 15 h of daily wear to achieve the desired expansion of approximately 0.25 mm per week.

#### 8.5.2. Fixed Maxillary Expanders

Fixed maxillary expanders are designed to produce rapid skeletal maxillary expansion and are indicated for unilateral or bilateral transverse deficiencies. Fixed expanders may be anchored with bands (banded expanders), mini-implants/mini-screws, or acrylic blocks (bonded expanders). A bonded expander can be used in any dentition stage, provided adequate root support remains. Otherwise, a banded expander anchored to the first molars, or first molars and first premolars, is recommended in the late mixed dentition stage. In late adolescence, skeletal anchorage is a good treatment option to avoid dental tipping. Adults typically require surgically assisted maxillary expansion. For patients with sagittal deficiencies, RME may be combined with orthopedic sagittal traction using a Delaire’s mask.

Fixed maxillary expanders have two types of force delivery mechanisms: screw expansion or spring expansion. The Hyrax-Expander is the most frequently used screw expander, and the Quad-Helix is the most common spring expander. The Hyrax-Expander is indicated for severe skeletal deficiencies, with activation occurring once or twice daily. The range of expansion is 0.25 mm to 0.5 mm per activation until an overcorrection of approximately 25% is achieved. The Quad-Helix is less bulky, requires less frequent reactivation, and does not rely on patient cooperation for adjustments. Its indication is a mild skeletal deficiency. Both expander devices require a minimum retention period of at least 3 to 6 months (Figure 10).

##### Rationale

Maxillary constriction increases nasal airway resistance, often leading to mouth breathing and altered tongue posture, which can contribute to retroglossal airway narrowing (Figure 2). RME increases maxillary width, enlarges the nasal floor and cavity, reduces nasal resistance, and favors nasal breathing by expanding the nasomaxillary complex. Additionally, it improves tongue posture and enlarges the pharyngeal airway [134]. Several studies report significant reductions in AHI after RME [135,136]. In the short term, RME may help improve the quality of life for children with a narrow maxilla [137]. Additionally, improvements in behavioral disturbances, cognitive abilities, and nasal function were reported after RME in children affected by snoring or OSA [138,139]. An SR-MA concluded that RME might help eliminate predisposing factors to OSA [140].

Nevertheless, current evidence on RME for treating pediatric OSA is inconclusive [141]. While some studies suggest RME may improve snoring and quality of life in children with persistent symptoms after adenotonsillectomy, overall findings are limited by methodological weaknesses and lack of control groups [138]. Interceptive orthodontic treatments may offer benefits, but cannot yet be recommended as standalone therapies for OSA [142].

### 8.6. Maxillary Protraction Treatment (Facemask)

**Figure 11:**
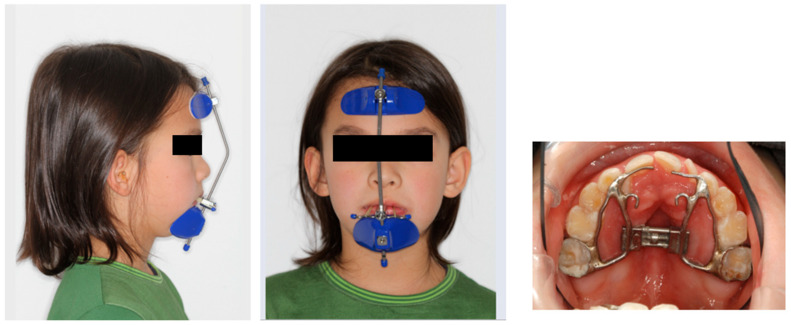
Protraction treatment: Extraoral (Delaire’s mask) and intraoral appliance (Photographs shown with parental and patient’s consent).

Retrusive maxilla and Class III malocclusion can result in a decreased retropalatal airway space [143]. Sagittal correction of the maxilla can be achieved through protraction treatment utilizing a Delaire’s mask (reverse pull headgear). The optimal time to initiate protraction of the retrognathic maxilla and to achieve the best results is between 6 and 9 years of age, before the fusion of the circummaxillary sutures. Early intervention allows for the mobilization of sutures and the maxillary complex and induces protraction osteogenesis [144]. If the patient is an adolescent, maxillary protraction can still be achieved through a miniplate-assisted facemask or maxillomandibular bone-to-bone traction techniques. On average, the maxilla can be protracted by approximately 5.5 mm (Figure 11). The most severely affected patients are often those with syndromic craniosynostosis, resulting in severe midfacial hypoplasia, where more aggressive approaches may be required.

#### Rationale

**Figure 12:**
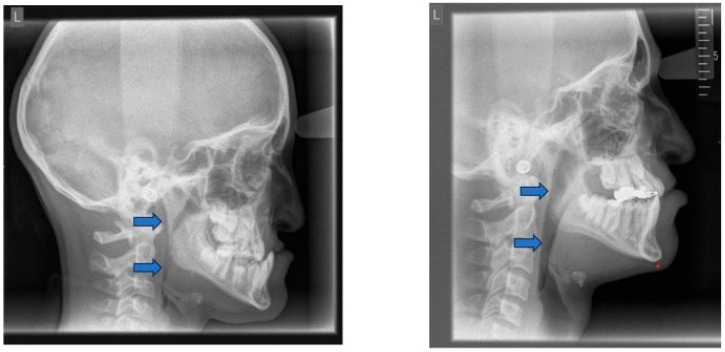
Posterior airway space (PAS) before (**left**) and after (**right**) protraction treatment. Blue arrows indicate the PAS.

Downward and forward growth of the maxilla can be stimulated by facemask therapy. The nasopharyngeal and velopharyngeal muscles, which function as upper airway dilators, are attached to the posterior nasal spine. Advancement of the maxilla displaces the posterior nasal spine in a forward and downward direction, thereby repositioning and activating these muscles. As a result, the tongue and soft palate are moved forward, contributing to an increase in the airway [145] (Figure 12). In children with skeletal Class III, particularly those between 7 and 12 years, there is evidence to support the positive impact of facemasks [146].

### 8.7. Functional Appliances for Class III Treatment

**Figure 13:**
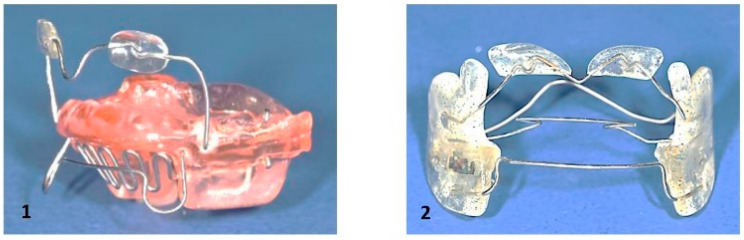
Functional Class III appliances: (**1**): Class III activator, (**2**): Fraenkel III.

Functional orthodontic appliances can be used for minor forms of maxillary retrognathia. Several functional appliances, such as the Fraenkel III or Class III activator, can guide and stimulate growth (Figure 13). Pads act as a shield, reducing the pressure exerted by the upper lip and the cheeks and applying traction on the periosteum (traction osteogenesis), thereby stimulating bone formation and anterior growth of the maxilla. These appliances are mainly used for retention after protraction treatment.

### 8.8. Appliance for Redirecting Vertical Growth Pattern

**Figure 14:**
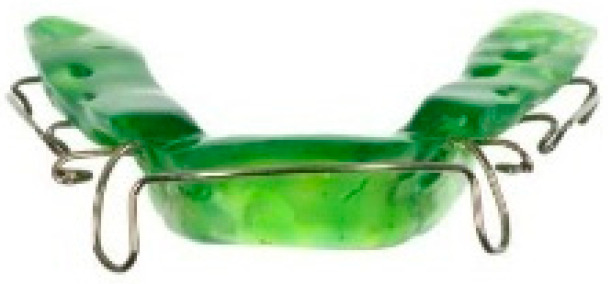
Bite plate for lower jaw.

A bite plate in the lower jaw is intended to mitigate the vertical growth pattern. This approach directs condylar growth in a more cranio-ventral direction, leading to a counterclockwise rotation of the mandible. However, the effect is typically subtle (Figure 14).

### 8.9. Mandibular Advancement

**Figure 15:**
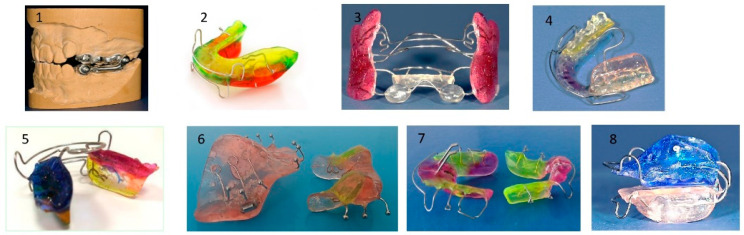
Most common appliances for mandibular advancement: (**1**) Herbst, (**2**) Activator, (**3**) Fraenkel II, (**4**) Bionator, (**5**) elastic activator, (**6**) Twin Block, (**7**) Bite-jumping appliance (VDP- Sander II appliance, (**8**) Activator (modification).

A retrognathic mandible induces retrodisplacement of the tongue, thereby reducing the upper airway volume. Orthopedic mandibular advancement was first introduced by Dr. Kingley with the “bite-jumping” appliance in 1879 [147]. This type of appliance aims to correct skeletal mandibular retrognathia by redirecting mandibular growth into a more forward and downward position, either passively or actively, while being worn either fixed or removably (for approximately 15 h per day). There are many functional Class II appliances, such as the classical activator and its modifications, the Fraenkel II appliance, the Herbst appliance, the Bionator, the Twin Block, and the bite-jumping appliance (VDP-Sander II appliance). In addition, some can be combined with maxillary expansion (e.g., Herbst-RME) appliances. All devices share the common goal of positioning the mandible into the desired therapeutic position, provided they are worn consistently until the intended orthopedic effect is achieved (Figure 15).

Dental and bony changes associated with the use of functional appliances in growing patients are well-documented [148,149]. Wearing functional appliances during growth is associated with increased mandibular length (long-term effect). The anticipated mandibular advancement ranges from approximately half to one premolar cusp. The advancement treatment phase typically lasts 6 to 9 months.

#### Rationale

As the tongue connects directly to the mandible, forward displacement of the mandible moves the tongue anteriorly and increases the retroglossal airway space. A more anterior tongue position leads to a more anterior position of the velum, which touches the tongue’s dorsum and opens the airway. In addition, forward displacement of the mandible decreases the collapsibility of the pharynx. The lateral wall of the soft palate connects to the base of the tongue through the palatoglossal arch. Mandibular advancement stretches the soft palate through the palatoglossal arch, stiffens the velopharyngeal segment (immediate effect) [150], and expands the pharynx in all three dimensions, especially the minimal cross-sectional area (MCA) of the pharynx [151].

An SR-MA stated that early treatment with functional appliances in growing patients with skeletal Class II malocclusions had positive effects on the upper airway, particularly on the oropharyngeal airway [152]. In a further systematic review, it was shown that various functional appliances (e.g., Herbst, Twin Block, MARA, Monobloc) improve upper airway volume in orthodontic patients (aged <18 years) after treatment [153]. Nevertheless, the current evidence on functional appliances and OSA is insufficient due to small sample sizes, lack of control groups, inadequate randomization, and the absence of long-term data [154].

### 8.10. Evidence of the Four Major Treatment Modalities

This section summarizes the available evidence supporting four primary orthodontic treatment modalities used in managing pediatric OSA: RME, maxillary protraction treatment (facemask therapy), mandibular advancement, and myofunctional therapy. The evidence of each treatment modality is presented based on recent SR-MAs:

- RME

An SR-MA including nine studies (172 patients) demonstrated that RME significantly reduces the AHI and increases the oxygen saturation (SPO_2_) in children with OSA. Nonetheless, the lack of control groups in most included studies limits the strength of the conclusions [155].

An additional comprehensive SR-MA, including 15 RME studies as part of a broader synthesis (25 studies in total), reinforced these findings, showing consistent AHI reduction and improvement in minimum SPO_2_. Nevertheless, the predominance of uncontrolled study designs underscores the need for further high-quality trials [142].

- Maxillary Protraction/Facemask Therapy

An SR-MA on growing Class III patients with maxillary retrognathia reported that maxillary protraction significantly increased the nasopharyngeal airway dimensions. The quantitative analysis included six high-quality studies, comprising 168 subjects and 140 untreated controls [145]. Although the analysis focused on morphologic airway changes rather than on functional respiratory outcomes, such as AHI, it supports potential benefits in selected patients.

- Mandibular Advancement Appliances

An SR-MA, including seven studies (four randomized controlled trials (RCTs) and three prospective non-RCTs), supports evidence for the use and efficacy of mandibular advancement appliances in pediatric OSA. Two high-quality RCTs contributed to the quantitative analysis, demonstrating significant reductions in AHI. Subgroup analysis suggested that this intervention was more effective in patients treated before the end of their pubertal growth spurt [156]. These results are further supported by the broader SR-MA [142], which also demonstrated significant improvements in AHI following mandibular advancement therapy.

- Myofunctional Therapy

A meta-analysis including ten studies (three RCTs, one case report, three prospective case series, two retrospective case series, and one prospective case-controls), involving 241 treated patients and 44 controls, concluded that myofunctional therapy (both active and passive) can reduce AHI, increase mean oxygen saturation, and significantly improve mouth breathing in children with mild to moderate OSA [157].

- Interpretation of Evidence and Clinical Implications

Across all four modalities, the available evidence suggests beneficial effects on key respiratory and functional outcomes in pediatric OSA. Among them, RME (15 studies) and mandibular advancement (5 studies) demonstrated the most consistent results in terms of AHI reduction. Nevertheless, most studies remained small in scale and duration, and often lacked control groups [142]. While interceptive orthodontics appears promising, the overall quality of evidence remains low to moderate. Consequently, these treatments cannot yet be considered definitive or elective therapies for pediatric OSA. Instead, their use should be guided by individualized orthodontic assessment within a multidisciplinary framework.

## 9. Limitations and Contraindications in Orthodontic Treatment for Pediatric OSA

### 9.1. Extractions

Orthodontic interventions, such as tooth extractions and distalization (moving teeth backward) that alter the anterior–posterior dimensions of tongue space, have been blamed for potentially exacerbating the risk of OSA. Thus, premolar extractions and bimaxillary retraction can decrease the oral cavity volume, restrict tongue space, and lead to posterior tongue displacement, hence resulting in a constriction of the upper airway, especially in the glossopharyngeal region.

A study compared changes in the PAS after treatment of Class II anomalies in patients with normodivergent and hyperdivergent facial patterns, both with and without premolar extractions. The most pronounced airway reduction was observed in the hyperdivergent extraction group, although it was not statistically significant [158].

A systematic review reported that in bimaxillary protrusion cases, premolar extraction followed by anterior retraction reduced upper airway dimensions. In contrast, space closure through molar mesialization was associated with increased airway volume, suggesting that anterior retraction may restrict the airway, while molar mesialization appears to preserve or even enlarge the airway [159]. 

In further systematic reviews [153,160] and meta-analysis [161], no substantial evidence has been reported linking dental extractions to airway space restriction. Nevertheless, it is essential to note that the patients included had a mean age of 19.1 years and had long passed the peak of craniofacial growth. In growing individuals, there are indications that tooth extractions result in reduced jaw growth and decreased airway space [162]. Given the established role of each tooth bud in promoting jaw growth, tooth extractions should be avoided before the growth spurt to achieve a maximum of intraoral volume to accommodate the tongue intraorally. Serial extractions should also be viewed critically in this context.

### 9.2. Headgear and Maxillary Growth Restriction

**Figure 16:**
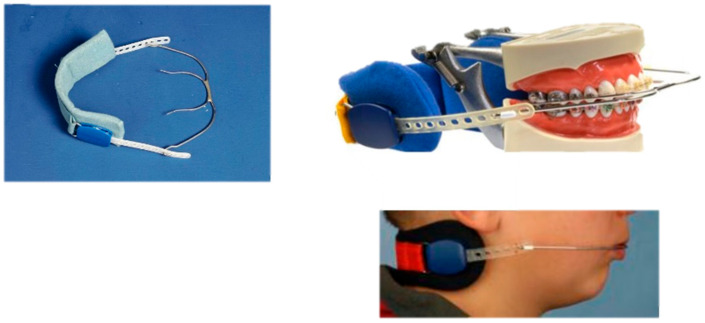
Headgear. The appliance is delivering intraoral traction forces, and it is anchored around the neck.

Headgear treatment is used for skeletal Class II correction by affecting the anterior–posterior position of the maxilla. Cervical headgear, used for at least 12 h daily with an average force of 450 g per side for one year, can effectively restrict anterior maxillary growth [163,164,165,166,167] (Table 1). This restriction may have an adverse effect on the dimensions of the nasopharyngeal airway and the intraoral tongue space. Children receiving combined activator-headgear treatment for skeletal Class II correction presented a significant negative impact on sagittal development of the maxilla (reduction of SNA angle: angle defined by nasion-sella-point A), when compared to controls without headgear treatment [168]. Thus, treatment with headgear may contribute to alterations in the dimensions of the nasopharyngeal and oropharyngeal airway [169]. A further study concluded that headgear treatment may even contribute to OSA during nighttime wear. Therefore, headgear treatment in children with retrognathic mandibles should be carried out with caution. If a patient already presents with OSA, headgear treatment may aggravate OSA [170] (Figure 16).

### 9.3. Craniofacial Adverse Effects of Continuous Positive Airway Pressure (CPAP) Therapy

Even if there are clear benefits to CPAP therapy, when it is implemented at a very young age, flattening of the midface or maxillary retrusion due to prolonged mask pressure may occur, and it must be carefully monitored [126,171]. This may reduce nasopharyngeal airway and intraoral volume, leading to a lower tongue (rest) posture and potentially affecting the orofacial development. Additionally, unwanted tooth movement is a further side effect of CPAP therapy, which can impact quality of life [172].

These dental and skeletal side effects may impact both function and aesthetics, highlighting the need for orthodontic monitoring in children undergoing prolonged CPAP therapy.

## 10. Discussion

### 10.1. Clinical Implications and Future Implementation Strategies

This review highlights the multifactorial pathophysiology of OSA, emphasizing the complex interplay of craniofacial anatomy, neuromuscular dysfunction, and upper airway patency that contributes to disease onset and progression [3,8]. The craniofacial features of OSA patients typically include mandibular retrognathia, maxillary constriction, a high-arched palate, vertical growth patterns, and reduced airway volume [28,81,82,83,84,85,86,87]. Myofunctional disorders are associated with mouth breathing, tongue dysfunction, and open-mouth posture [57,61,70,71], which can further exacerbate upper airway obstruction and contribute to the heterogeneity of clinical presentations.

Despite the relatively high prevalence of OSA in specific high-risk groups, particularly those with craniofacial syndromes [38,39], the condition remains underdiagnosed in pediatric populations due to the lack of standardized diagnostic protocols, small cohorts in observational studies, low clinical awareness, and heterogeneous diagnostic criteria.

Orthodontists are strategically well-positioned to identify craniofacial risk factors and implement interceptive orthopedic measures, contributing to the early detection, risk stratification, and intervention in the interdisciplinary management of pediatric OSA [14,100,173]. Early orthodontic screening enables the timely identification of anatomical and functional risk factors [99], allowing for targeted interventions that can alter growth trajectories, mitigate airway obstruction, and potentially reduce the need for future airway-improving orthognathic surgery.

Orthodontic therapies, including RME, mandibular advancement, and maxillary orthopedic protraction, offer preventive and therapeutic benefits by addressing underlying skeletal discrepancy and orofacial dysfunction and thereby optimizing skeletal development [140,142]. These treatment interventions further contribute to enlarging the upper airway space. Therefore, these approaches should be carefully planned in close collaboration with pediatricians, sleep specialists, otolaryngologists, and speech-language pathologists, forming an integrated multidisciplinary approach. Treatment planning should consider the child’s age, developmental status, and ability to comply with the treatment regimen. Importantly, parental and caregiver education should accompany clinical interventions to enhance adherence and outcomes.

Myofunctional therapy [122,123], both active (e.g., orofacial exercises) and passive (e.g., intraoral appliances) [127,128], has emerged as a valuable non-invasive adjunctive strategy for improving airway patency and respiratory parameters, as well as enhancing sleep quality and daytime function [125]. However, success depends heavily on patient cooperation and individualized treatment planning [126]. Orthodontists are well-equipped to deliver and monitor these therapies as part of a comprehensive airway-centered treatment strategy.

According to the American Association of Orthodontists 2019 White Paper, the primary role of orthodontists in managing OSA is to screen for signs, recognize potential cases, and refer patients to appropriate physician specialists for diagnosis and treatment [100]. Orthodontic treatment may support OSA management as part of a multidisciplinary team, but it should begin only after a thorough medical evaluation has been conducted. The diagnosis of OSA lies outside the orthodontist’s scope. Therapeutic interventions, such as palatal expansion or mandibular advancement devices, should only be considered when supported by clear clinical indications, effectiveness, and potential side effects [14]. Screening children for OSA should be an integral part of the orthodontic assessment, as a high risk of OSA has been identified in nearly 30% of children undergoing orthodontic treatment [174].

Importantly, even after adenotonsillectomy, the current first-line surgical approach, sleep-disordered breathing frequently persists, especially in patients with underlying craniofacial risk factors. This observation further highlights the importance of orthodontic assessment and intervention in comprehensive care pathways for pediatric OSA.

Young patients with craniofacial risk factors may experience a more substantial impact [61,68,69,70,71,72]. RME is a key intervention that widens the nasal cavity, reduces nasal resistance, and improves tongue posture and breathing [134,135,136,137,138,139,140].

While interventions like RME or mandibular advancement have been proposed, robust long-term evidence is lacking. Integrating dentists and orthodontists into the multidisciplinary teams is essential to improving the recognition and management of pediatric OSA [173], thereby reducing the burden of delayed diagnosis. Furthermore, early orthodontic assessment combined with timely sleep evaluation may facilitate earlier detection across populations, underscoring the need for more preventive care strategies.

In addition, optimizing care pathways, standardizing diagnostic protocols [99]—particularly by incorporating routine airway assessments during orthodontic examinations—and establishing clear referral guidelines are crucial steps towards enhancing early detection and treatment outcomes. Orthodontists should be integrated into pediatric OSA care networks for treatment, prevention, and long-term follow-up.

Moreover, this review highlights current limitations in the literature, including the scarcity of long-term data on orthodontic interventions in pediatric OSA and the considerable variability in diagnostic criteria and treatment protocols.

### 10.2. Research Implications and Future Implementation Strategies

Despite the growing clinical interest and promising short-term outcomes, the long-term efficacy and stability of orthodontic interventions for OSA remain insufficiently characterized. Existing studies are often limited, with varying diagnostic criteria, treatment protocols, heterogeneous study populations, inconsistent follow-up periods, and a lack of long-term data. This variability compromises the ability to draw definitive conclusions and translate findings into standardized care models.

Future research should focus on prospective, multicenter trials that evaluate the efficacy of combined therapeutic approaches across diverse populations, particularly in growth outcomes and quality-of-life measures. Inconsistent diagnostic and treatment protocols underline the need for standardized longitudinal research.

Collaborative research is crucial for developing standardized and personalized treatment protocols, as well as enhancing long-term outcomes [175,176]. High-quality prospective trials should evaluate the long-term impact of combined therapeutic approaches on airway function, craniofacial growth, and overall quality of life across diverse pediatric populations [177].

This narrative review provides a foundation for future research, highlights the need for evidence-based clinical practice, and identifies knowledge gaps regarding prevention, early detection, and evolving needs of pediatric patients with OSA.

## 11. Conclusions

Orthodontists play an integral role in the interdisciplinary management of pediatric OSA through the early identification of craniofacial and functional risk factors and the implementation of growth-modifying interventions, thereby supporting airway expansion. Frequently co-existing myofunctional disorders should also be addressed to ensure treatment success. These interventions, when carefully selected and executed within a multidisciplinary framework, may not only support favorable craniofacial development but also serve as a preventive strategy against adult-onset OSA.

Pediatric OSA, if left untreated, carries significant consequences ranging from neurocognitive impairments to behavioral, cardiovascular, and metabolic dysfunction.

Orthodontic treatment offers the potential to modify craniofacial growth, optimize tongue posture, and improve airway patency through orthopedic interventions such as RME, mandibular advancement, and myofunctional therapy.

Special attention must be given to patients with mouth breathing, snoring, open-mouth posture, low tongue position, lateral crossbite, long face, and mandibular retrognathism. Additionally, children with craniofacial syndromes carry an increased risk for OSA and require early recognition and closer monitoring. After growth completion, correction of skeletal discrepancies in patients with OSA can only be achieved through orthognathic surgery.

However, orthodontic therapies must be guided by robust clinical evidence, clear indications, and collaborative care to avoid treatments where long-term benefits remain unproven. The implementation of early screening and intervention must carefully balance the potential benefits with the current limitations of available evidence to prevent overexertion of orthodontic practices in the absence of long-term data.

Continued interdisciplinary research, collaborative care models, and high-quality clinical trials are indispensable to refining treatment protocols, optimizing patient outcomes, and advancing precision medicine approaches for pediatric OSA.

In summary, pediatric OSA is a multifactorial condition that often differs markedly from adult presentation in both etiology and clinical manifestations. Early identification and age-appropriate, individualized treatment strategies are crucial for preventing long-term developmental, behavioral, and systemic consequences.

Orthodontic interventions, such as RME, mandibular advancements, and myofunctional therapy, demonstrate promising and still evolving evidence in managing pediatric OSA.

Close collaboration between medical and dental specialties remains essential to ensure accurate diagnosis, timely treatment, and effective care delivery for children with sleep-disordered breathing.

## Figures and Tables

**Table 1:** Cervico-craniofacial skeletal (**a**) and soft tissue morphology (**b**) in patients with OSA.

**(a) Skeletal Variables**
Shorter dimension of the cranial base (distance of S-N decreased) Shorter maxillary and mandibular lengths Slight counter-clockwise rotation of clivus (angle N-S-Ba decreased) Pronounced maxillary and mandibular retrognathia Increased anterior facial height and mandibular plane angle (defined by cranial base and mandibular plane) Increased gonion angle Deviated head posture with a larger cranio-cervical angle (hyperextension of cervical spine)
**(b) Soft Tissue Variables**
Increased length and thickness of the soft palate Increased sagittal area of the tongue Caudally extended tongue mass Decreased sagittal dimensions: ○ nasopharynx (due to adenoids) ○ velopharynx (due to soft palate) ○ oropharynx with reduced distance between the base of the tongue and the posterior pharyngeal wall (due to tongue)

**Table 2:** Extraoral and intraoral findings in children with OSA.

Extraoral Findings	Intraoral Findings
Adenoid facies: ○ Dark circles around the eyes ○ Flattened cheekbones ○ Dry lips ○ Labial incompetence ○ Underdeveloped nasal bones ○ Pronounced nasolabial furrows ○ Open mouth posture ○ Reduced orofacial muscle tonicity ○ Long-face	Narrow maxilla Higher and narrower palatal vault Posterior crossbites Open bite or deep bite Increased overjet Proclined upper incisors Low tongue position Ankyloglossia/reduced tongue mobility
Convex profile	
Retrognathic mandible	
Increased mandibular angle Head in hyperextension	

**Table 3:** Intraoral and extraoral typical features of “at-risk” children.

Typical Features of an “At-Risk Patient”
Snoring
Mouth breathing
Open mouth posture
Long-face
Narrow maxilla with lateral crossbites and high-arched palate
Increased overjet/open bite
Retrognathic mandible
Muscle hypotonia

## Abbreviations

AHI	Apnea-Hypopnea index
AI	Apnea index
ANS	Anterior nasal spine
Ba	Basion
CBCT	Cone-beam computed tomography
CPAP	Continuous positive airway pressure
DISE	Drug-induced sleep endoscopy
DOME	Distraction osteogenesis maxillary expansion
MARPE	Mini-implant/mini-screw-assisted palatal expansion
MCA	Minimal cross-sectional area
N	Nasion
OSA	Obstructive sleep apnea
PAS	Posterior airway space
PNS	Posterior nasal spine
PSQ	Pediatric Sleep Questionnaire
RCT	Randomized controlled trials
RME	Rapid maxillary expansion
SARPE	Surgically assisted palatal expansion
SR-MA	Systematic review and meta-analysis
S	Sella
SN-GoGn	Angle formed by sella-nasion to gonion-gnathion
TPP	Tübingen palatal plate
TRMR	Tongue Range of Motion Ratio
